# University physical education teacher leadership behaviors and college students’ learning outcomes: the mediating roles of basic psychological need satisfaction and frustration

**DOI:** 10.3389/fpsyg.2026.1858254

**Published:** 2026-07-16

**Authors:** Liqiu Huang, Kyung Ho Kim, Hyun-Duck Kim, Angelita Bautista Cruz

**Affiliations:** 1 College of Physical Education, Keimyung University, Daegu, Republic of Korea; 2 Department of Business Administration, Keimyung University, Daegu, Republic of Korea; 3 Department of Sport Marketing, Keimyung University, Daegu, Republic of Korea; 4 Department of Kinesiology, Keimyung University, Daegu, Republic of Korea

**Keywords:** basic psychological needs, boredom, class satisfaction, learning motivation, multidimensional model of leadership, PE teacher leadership behaviors, self-determination theory

## Abstract

**Introduction:**

Grounded in the Multidimensional Model of Leadership (MML) and Self-Determination Theory (SDT), this study examines how physical education (PE) teacher leadership behaviors shape college students’ learning experiences, with a particular focus on the mediating mechanisms of basic psychological need satisfaction and need frustration.

**Methods:**

Data were collected via questionnaires from 504 undergraduate students enrolled in compulsory PE courses across several Chinese universities. Students’ perceived leadership behaviors of PE instructors, basic psychological need satisfaction and frustration, learning motivation, class satisfaction, and boredom were measured using the following questionnaires: Physical Education Teacher Leadership Behavior, Basic Psychological Need Satisfaction and Frustration (BPNSFS), Perceived Locus of Causality, Physical Education (PACSQ), and class boredom.

**Results:**

Structural equation modeling revealed that need satisfaction and need frustration served as parallel mediators of the relationship between PE teacher leadership and three key learning outcomes: learning motivation, class satisfaction, and class boredom. Leadership behaviors exhibited fully mediated effects on learning motivation and boredom, and partially mediated effects on class satisfaction. Furthermore, the mediating pathway through need satisfaction demonstrated a significantly stronger overall effect than that through need frustration.

**Conclusion:**

These findings indicate that PE teachers influence students’ experiences primarily by fulfilling or thwarting basic psychological needs, alongside a direct positive contribution to class satisfaction. This study provides robust empirical support for integrating leadership and motivational theories, and offers actionable insights for enhancing pedagogical practices in higher education PE settings.

## 1 Introduction

Higher education has increasingly confronted challenges such as declining classroom engagement, fluctuating student motivation, and widening disparities in learning experiences (Halabieh et al., 2022). These concerns are particularly pronounced in physical education (PE) contexts, where insufficient physical activity participation and reduced classroom engagement among university students have attracted growing scholarly attention (Kljajević et al., 2021; Brown et al., 2024). In the Chinese context, national policies and empirical surveys have similarly documented unstable participation patterns, fluctuating classroom motivation, and deteriorating learning experiences among university students (Liu et al., 2022; Pan et al., 2025; Xu et al., 2025). These patterns suggest that curricular content and instructional design alone may be inadequate to sustain students’ motivation for PE participation. Consequently, social-contextual factors—particularly teachers’ instructional behaviors and leadership styles—have been increasingly recognized as critical determinants of student motivation and classroom experience (Kim and Cruz, 2024).

Recent literature further highlights that effective teacher leadership can directly mitigate academic burnout and disengagement by proactively satisfying students’ basic psychological needs (Ryan and Deci, 2020; Kim and Cruz, 2024). In PE settings, teachers are responsible not only for delivering movement knowledge and skills but also for organizing activities, managing learning environments, ensuring student safety, and motivating participation. Owing to the unique activity-based and highly interactive nature of PE classes, teachers’ leadership behaviors are particularly influential in shaping students’ psychological needs, motivation, and learning experiences (Chelladurai and Saleh, 1980; Kim and Cruz, 2024; Vasconcellos et al., 2020). Research grounded in Self-Determination Theory (SDT) has demonstrated that teachers’ supportive behaviors can enhance students’ classroom engagement, motivational quality, and overall well-being (Reeve and Cheon, 2021; Yun et al., 2024). For example, Behzadnia et al. (2018) found that students’ perceptions of teacher autonomy support were positively associated with psychological need satisfaction, autonomous motivation, well-being, and intentions for continued participation. Despite this progress, existing research has predominantly focused on the positive effects of supportive teaching behaviors, while comparatively less attention has been devoted to understanding how controlling or neglectful leadership behaviors contribute to students’ negative experiences through underlying psychological mechanisms (Tian and Shen, 2023; Viksi and Tilga, 2022).

Moreover, the processes through which teacher behaviors translate into students’ motivational and emotional experiences remain insufficiently integrated within a coherent theoretical framework (Aelterman et al., 2019; Noetel et al., 2023). SDT offers a comprehensive framework for understanding how the classroom social environment influences the quality of students’ motivation and learning experiences (Deci and Ryan, 2000; Ryan and Deci, 2020). In PE settings, a substantial body of empirical evidence has demonstrated that basic psychological needs play a key mediating role in linking teacher behaviors to outcomes such as learning motivation, class satisfaction, and behavioral intentions (Vasconcellos et al., 2020; Yun et al., 2024).

In the present study, learning experiences are conceptualized as a broad outcome construct reflecting students’ overall psychological and emotional experiences in physical education classes. Specifically, learning motivation, class satisfaction, and boredom are regarded as key dimensions of learning experiences, representing students’ motivational, evaluative, and emotional responses to the learning environment, respectively. Examining these dimensions simultaneously provides a more comprehensive understanding of how students experience and respond to university physical education classes.

Simultaneously, the Multidimensional Model of Leadership (MML) emphasizes that leadership effectiveness depends on the degree of congruence among situational demands, leader behaviors, and individual characteristics (Chelladurai, 2007, 2014). This model provides a valuable theoretical lens for examining the structural features of PE teachers’ behaviors. However, prior MML-based research has largely focused on direct associations between different leadership behavior types and performance-related outcomes. Considerably less is known about how teacher leadership behaviors shape students’ learning experiences through internal psychological mechanisms (Aelterman et al., 2019; Vansteenkiste et al., 2020).

Accordingly, the present study is situated within the context of university PE classes in China and integrates the MML with SDT. Specifically, it proposes a dual-path mediation model in which PE teacher leadership behaviors serve as antecedents, psychological need satisfaction and need frustration function as parallel mediators, and learning experiences constitute the outcome variables. Compared with previous studies, the present research contributes to the literature in several ways. First, it extends existing knowledge by examining PE teacher leadership behaviors within Chinese university physical education classes, a context that has received considerably less attention than competitive sport settings, coaching environments, and primary and secondary school PE. Second, it integrates the Multidimensional Model of Leadership and Self-Determination Theory into a unified framework, thereby providing a more comprehensive explanation of how teacher leadership behaviors influence students’ learning outcomes through psychological processes. Third, unlike previous studies that have primarily focused on positive outcomes, the present study simultaneously examines both need satisfaction and need frustration and incorporates learning motivation, class satisfaction, and boredom into the same model, allowing for a more balanced understanding of both positive and negative learning experiences in university PE settings. Therefore, this study extends the application of SDT within higher education PE settings and provides a psychological, process-oriented explanation for the MML. In doing so, it offers empirical evidence for understanding of leadership effects in education in general and university PE classrooms in particular.

## 2 Theoretical foundations and integrated models

### 2.1 The structural significance of the multidimensional model of leadership in physical education classrooms

Chelladurai’s Multidimensional Model of Leadership (MML) provides a foundational framework for understanding the relationship between leadership behavior and individual outcomes (Chelladurai, 1980). The model proposes that leadership effectiveness depends on the congruence among required behavior, actual leader behavior, and preferred behavior of group members (Chelladurai, 2007). This matching structure underscores that leadership behavior does not operate in isolation; rather, its effects vary according to specific situational characteristics and individual differences, thereby influencing members’ satisfaction and performance (Kim and Cruz, 2024).

The Leadership Scale for Sports (LSS), developed by Chelladurai and Saleh (1980), provided an essential operational tool for the MML and enabled the theory to be systematically examined in sport settings. Subsequent studies have continued to test the model across cultures and adapt it to different contexts (Solera-Alfonso et al., 2025; Teques et al., 2021). In the Chinese context, Zhang (2021) empirically validated and refined the five-dimensional structure of leadership behavior based on the organizational characteristics and teaching culture of university PE classes. The revised dimensions include training and instruction, social support, management, recognition, and communication, making the framework more closely aligned with the pedagogical logic of university PE instruction. Accordingly, the present study adopts these five dimensions to define the structure of PE teacher leadership behavior.

### 2.2 Self-determination theory and dual-path psychological mechanisms

Self-Determination Theory (SDT) posits that the quality of individuals’ motivation depends on the extent to which three basic psychological needs—autonomy, competence, and relatedness—are satisfied (Deci and Ryan, 2000; Vansteenkiste et al., 2020). When the social environment supports these psychological needs, individuals are more likely to develop high-quality forms of motivation, such as intrinsic motivation and identified regulation (Vansteenkiste et al., 2020). Conversely, when the environment is controlling, coercive, or neglectful, it may lead to need frustration and subsequently result in negative outcomes such as anxiety, amotivation, emotional withdrawal, and reduced engagement in learning activities (Tian and Shen, 2023).

Building on this perspective, recent developments in SDT have further distinguished between the dual psychological processes of basic psychological need satisfaction (BPNS) and basic psychological need frustration (BPNF; Bhavsar et al., 2020). Research has demonstrated that need frustration is not merely the absence of need satisfaction; rather, it reflects a distinct damaging psychological experience triggered by controlling or need-thwarting social environments and possesses unique predictive power for negative emotions and maladaptive functioning (Vansteenkiste et al., 2020). This dual-path perspective provides a more systematic theoretical basis for understanding the two-sided psychological effects of teacher behavior in PE classrooms.

### 2.3 Integration of the multidimensional leadership behavior model and self-determination theory

The MML provides a structural framework for understanding PE teachers’ leadership behaviors in classroom settings, with a particular emphasis on what teachers do. The model suggests that teacher leadership is not a unidimensional construct but rather a combination of multiple behavioral dimensions—such as training and instruction and social support—that may vary across instructional contexts and student characteristics (Chelladurai, 2014; Teques et al., 2021). In this sense, the model is useful for identifying specific types of leadership behaviors displayed by PE teachers and for providing a behavioral basis for examining their associations with student outcomes.

SDT, in turn, helps explain why teacher behaviors exert influence. According to SDT, the social environment does not directly determine students’ motivation, emotions, or behavioral outcomes; rather, its effects are transmitted through the extent to which students experience satisfaction or frustration of their basic psychological needs (Ryan and Deci, 2020). In PE classes, supportive teacher behaviors may foster students’ autonomy, competence, and relatedness need satisfaction, thereby promoting higher-quality motivation and more positive classroom experiences. By contrast, controlling or negative teacher behaviors may contribute to need frustration, which may in turn undermine motivational quality and increase negative classroom experiences (Vansteenkiste et al., 2020). SDT therefore provides a process-oriented explanation for how teacher behaviors become linked to student outcomes.

**Figure 1:**
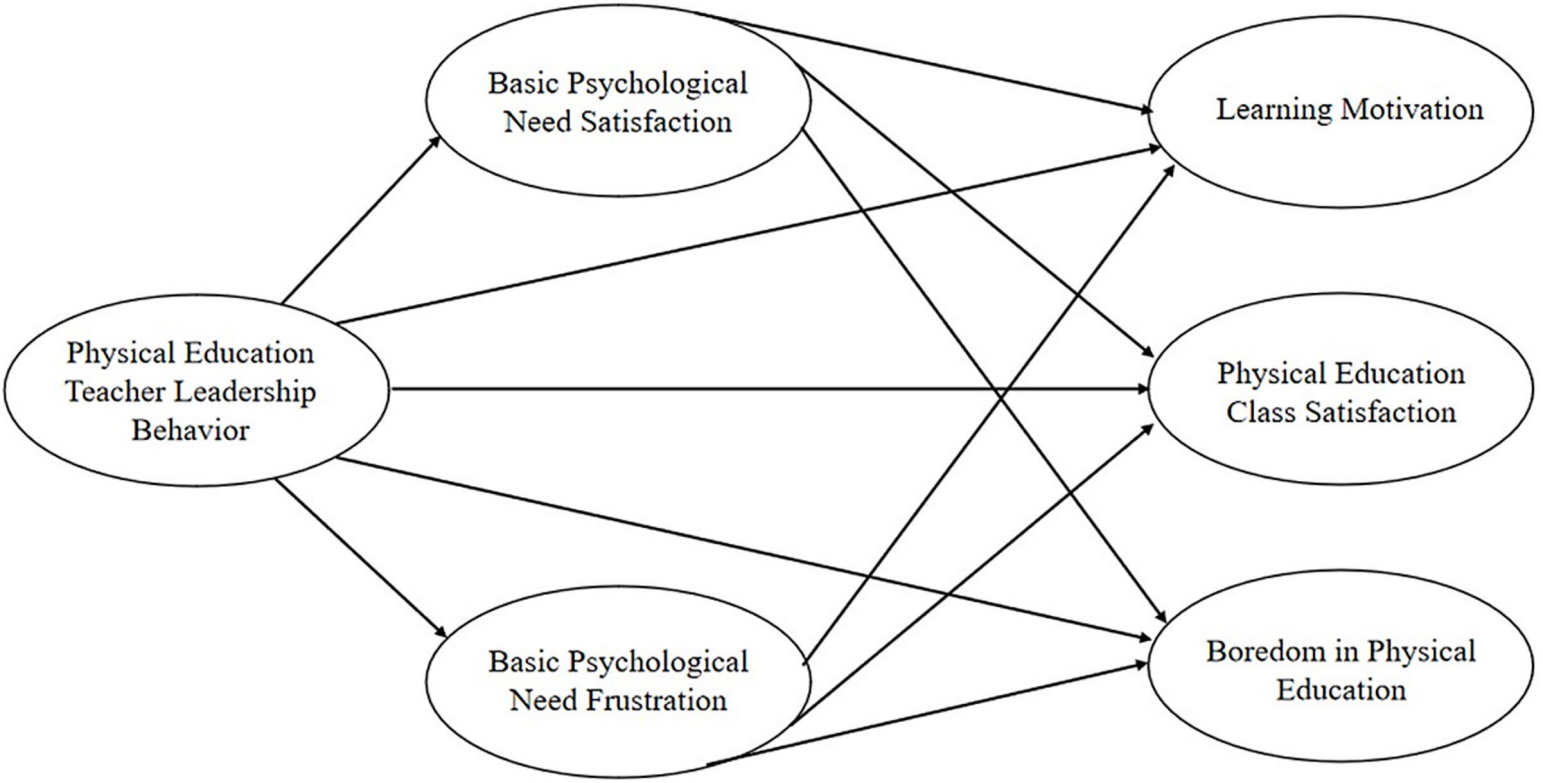
Conceptual mediation model of PE teacher leadership, basic psychological need satisfaction and frustration, and learning outcomes.

Conceptualizing PE teacher leadership behavior as a contextual antecedent, basic psychological need satisfaction and need frustration as parallel mediating processes, and learning experiences as outcome variables allows for a more integrated and explanatory framework (Figure 1). Such an approach makes it possible to examine not only whether teacher leadership behavior is associated with student outcomes, but also how these associations operate through students’ psychological need-based experiences.

Accordingly, the present study was designed to address three interrelated questions. First, does PE teacher leadership behavior predict students’ basic psychological need satisfaction and need frustration? Second, is PE teacher leadership behavior associated with students’ learning motivation, class satisfaction, and boredom? Third, do basic psychological need satisfaction and need frustration mediate the relationship between PE teacher leadership behavior and students’ learning experiences (learning motivation, class satisfaction, and boredom)?

## 3 Methods

### 3.1 Participants

A total of 504 university students volunteered to participate in the study. There were 172 males (34.1%) and 332 females (65.9%). All participants were non-PE majors ranging in age from 18 to 20 years: 170 participants were 18 years old (33.7%), 221 were 19 years old (43.8%), and 113 were 20 years old (22.4%). There were 260 first year (51.6%) and 244 second year students and they were majority taught by female instructors (*n* = 414, 82.1%). Participants were recruited from several regions across China such as Xinjiang, Henan, Shaanxi, Liaoning, Guizhou.

### 3.2 Measurement tools

#### 3.2.1 Physical education teacher leadership behavior

PE teacher leadership behavior was assessed using the scale revised by Zhang (2021), developed based on the MML. The 38-item scale consists of five dimensions: training and instructional behavior (e.g., My PE teacher carefully guides each student on movement techniques during practice), social support behavior (e.g., My PE teacher proactively checks on my progress), management behavior (e.g., My PE teacher strictly requires me to follow class rules), recognition behavior (e.g., My PE teacher praises me publicly for my good performance), and communication behavior (e.g., My PE teacher encourages me to fully express my ideas during discussions) Students were asked to rate how frequently their PE teachers demonstrated leadership behaviors during PE classes. Responses were rated on a 5-point Likert scale ranging from 1 (never) to 5 (always). The internal reliability scores for this study were 0.905, 0.929, 0.890, 0.907, and 0.912 for training and instructional behavior, social support behavior, management behavior, recognition behavior and communication behavior, respectively. The overall Cronbach’s alpha coefficient of the scale was.974.

#### 3.2.2 Basic psychological need satisfaction and frustration

Basic psychological need satisfaction and frustration of students were measured using the BPNSFS scale developed by Chen et al. (2015) adapted in PE context. This 24-item scale consists of 6 dimensions to separately assess satisfaction and frustration across the three basic psychological needs of autonomy, competence, and relatedness. Students were requested to report their feelings of satisfaction and frustration related to their PE class using a 5-point Likert scale ranging from 1 (not at all true) to 5 (completely true). The internal reliability scores for all dimensions in this study were 0.862 (autonomy satisfaction), 0.851 (relatedness satisfaction), 0.852 (competence satisfaction), 0.836 (autonomy frustration), 0.845 (relatedness frustration) and 0.855 (competence frustration). The overall Cronbach’s alpha coefficient of the scale was.849.

#### 3.2.3 Learning motivation

Students’ learning motivation in PE was measured using the Perceived Locus of Causality scale (PLOC-U; Sánchez de Miguel et al., 2017). The 20-item scale has five dimensions: intrinsic motivation, identified regulation, introjected regulation, external regulation, and amotivation. Responses were rated on a 6-point scale ranging from 1 (completely disagree) to 6 (completely agree). The internal reliability scores for this study were 0.859, 0.896, 0.854, 0.847, and 0.875 for intrinsic motivation, identified regulation, introjected regulation, external regulation, and amotivation, respectively. The overall Cronbach’s alpha coefficient of the scale was.843.

#### 3.2.4 Class satisfaction

PE class satisfaction was assessed using the Physical Education Class Satisfaction Scale (PACSQ) developed by Cunningham (2007). The 45-item scale consists of nine dimensions of satisfaction: mastery experiences, cognitive development, teaching, normative success, interaction with others, fun and enjoyment, improvement of health and fitness, diversionary experiences, and relaxation. Students were asked to report different sources of their satisfaction regarding their participation in physical activities during PE classes using an 8-point Likert scale ranging from 1 (completely dissatisfied) to 8 (extremely satisfied). The internal reliability scores for all dimensions in this study were 0.939 (mastery experience), 0.943 (cognitive development), 0.942 (teaching quality), 0.890 (normative success), 0.947 (interaction with others), 0.945 (fun and enjoyment), 0.945 (improvement in health and fitness), 0.933 (diversion experience) and 0.924 (relaxation). The overall Cronbach’s alpha coefficient of the scale was 0.990.

#### 3.2.5 Boredom

Class boredom was measured using a single-item indicator adapted from Reijseger et al. (2013), rated on a 5-point scale ranging from 1 (strongly disagree) to 5 (strongly agree): “In this semester’s physical education classes, I often feel bored.”

The measurement models demonstrated robust internal consistency and convergent validity (see Table 1). Cronbach’s alpha coefficients ranged from 0.836 to 0.947, composite reliability (CR) from 0.838 to 0.949, and average variance extracted (AVE) from.514 to.811, exceeding the recommended thresholds (Hair et al., 2019).

**Table 1:** Reliability and validity of each scale dimension.

Scale	Dimension	Factor loadings	Cronbach’s *α*	CR	AVE
TLB	TTB	0.595–0.844	0.905	0.903	0.540
	SSB	0.706–0.833	0.929	0.928	0.619
	AB	0.652–0.784	0.890	0.889	0.501
	RB	0.633–0.845	0.907	0.910	0.592
	CB	0.650–0.850	0.912	0.914	0.606
BPNS	AS	0.646–0.871	0.862	0.863	0.614
	RS	0.626–0.819	0.851	0.852	0.593
	CS	0.732–0.830	0.852	0.858	0.602
BPNF	AF	0.702–0.799	0.836	0.838	0.564
	RF	0.722–0.822	0.845	0.849	0.586
	CF	0.731–0.821	0.855	0.854	0.594
LM	IM	0.720–0.852	0.859	0.787	0.610
	IDR	0.782–0.855	0.896	0.899	0.690
	INTR	0.730–0.814	0.854	0.775	0.597
	EXTR	0.711–0.821	0.847	0.850	0.588
	AMO	0.774–0.828	0.875	0.798	0.638
PECS	ME	0.803–0.899	0.939	0.940	0.759
	CD	0.852–0.899	0.943	0.945	0.774
	TCH	0.858–0.898	0.942	0.941	0.761
	NS	0.746–0.842	0.890	0.891	0.621
	IWO	0.823–0.904	0.947	0.949	0.755
	FE	0.887–0.912	0.945	0.945	0.810
	IHF	0.868–0.897	0.945	0.946	0.778
	DE	0.686–0.892	0.933	0.936	0.711
	REL	0.815–0.898	0.924	0.923	0.749
BPE	BPE	–	–	–	–

TTB = training and instruction; SSB = social support; AB = management; RB = recognition; CB = communication; AS = autonomy satisfaction; RS = relatedness satisfaction; CS = competence satisfaction; AF = autonomy frustration; RF = relatedness frustration; CF = competence frustration; IM = intrinsic motivation; IDR = identified regulation; INTR = introjected regulation; EXTR = external regulation; AMO = amotivation; ME = mastery experience; CD = cognitive development; TCH = teaching quality; NS = normative success; IWO = interaction with others; FE = fun and enjoyment; IHF = improvement in health and fitness; DE = diversion experience; REL = relaxation. CR = composite reliability; AVE = average variance extracted.

Because all instruments were established and previously validated scales, the original response formats were retained to preserve their psychometric properties and facilitate comparison with prior studies.

### 3.3 Procedure

This study used a quantitative cross-sectional design, and the participants were recruited using a convenience sampling method from five universities located in different regions of China. Prior to the formal survey, the research team completed the translation and back-translation of the instruments and conducted a pilot test to ensure linguistic accuracy and cultural appropriateness of the measures. Data collection occurred from September 15 to September 30, 2025, recruiting undergraduate students from different universities across China. Because public PE is a compulsory course for first- and second-year students in Chinese higher education institutions (Wang, 2020), PE instructors assisted with participant recruitment by providing an initial introduction to the study during class. Given that the questionnaire consisted of 128 items and required approximately 15–20 min to complete, the first author prepared a pre-recorded video to ensure that all participants received standardized instructions. The video explained the purpose of the study, research procedures, data usage principles, participants’ rights, confidentiality protection, and the importance of participation. Before completing the questionnaire, all participants were required to read and provide informed consent electronically. Participation was entirely voluntary, and participants were informed that they could withdraw from the study at any time without penalty. After providing consent, participants completed the questionnaire anonymously by scanning a QR code linked to the Wenjuanxing platform. No personally identifiable information was collected during the survey process. All responses were treated as confidential and used solely for research purposes. The collected data were accessible only to the research team and were reported in aggregate form. This study was conducted in accordance with the ethical principles of the American Psychological Association (APA) and the Declaration of Helsinki. Ethical approval was obtained from the Institutional Review Board of Keimyung University, Republic of Korea (IRB No. 40525-202411-HR-071-03). Informed consent was obtained from all participants who volunteered in the study.

### 3.4 Data analysis

This study used SPSS 27.0 and AMOS 24.0 for data analysis. Descriptive statistics, reliability and validity tests (Table 2), and correlation analyses were first conducted for all study variables. Independent-samples *t*-tests were subsequently performed to examine gender differences. Finally, structural equation modeling was employed to test the path relationships among the variables. Mediation effects were estimated using 5,000 bootstrap resamples to calculate 95% bias-corrected confidence intervals, in accordance with the recommendations of Hayes (2022).

**Table 2:** Independent-samples *t*-tests comparing study variables by student sex.

Variables	Male		Female		*t*	*p*	Cohen’s *d*
	*M*	*SD*	*M*	*SD*			
TLB	4.48	0.55	4.41	0.50	1.30	0.195	0.13
BPNS	4.07	0.70	3.96	0.70	1.69	0.092	0.16
BPNF	2.34	1.07	2.12	0.90	2.30	0.022*	0.23
LM	4.53	0.65	4.37	0.57	2.67	0.008**	0.26
PECS	6.89	1.06	6.76	1.00	1.42	0.155	0.13
BPE	2.35	1.35	2.16	1.24	1.60	0.110	0.15

TLB = Teacher Leadership Behavior; LM = Learning Motivation; PECS = Physical Education Class Satisfaction; BPE = Boredom in Physical Education; BPNS = Basic Psychological Need Satisfaction; BPNF = Basic Psychological Need Frustration; *M* = mean, *SD* = standard deviation. Cohen’s *d* values of 0.20, 0.50, and 0.80 indicate small, medium, and large effects, respectively. *p < .05. **p < .01.

## 4 Results

### 4.1 Student sex differences

Independent-samples *t*-tests (Table 1) indicated that male students scored significantly higher than female students on basic psychological need frustration (*M* = 2.34 vs. *M* = 2.12) and PE motivation (*M* = 4.53 vs. *M* = 4.37). No significant gender differences emerged for PE teacher leadership behavior, basic psychological need satisfaction, class satisfaction, or class boredom (*p* > 0.05). Overall, gender differences were observed primarily in basic psychological need frustration and PE motivation, although the effect sizes were small (Lovakov and Agadullina, 2021).

### 4.2 Correlation among variables

As shown in Table 3, TLB was positively correlated with BPNS, LM, and PECS and negatively correlated with BPNF and BPE. According to Schober et al. (2018), the significant correlations ranged from weak to moderate in magnitude. TLB showed moderate positive correlations with BPNS (*r* = 0.637, *p* < 0.01) and PECS (*r* = 0.609, *p* < 0.01), whereas its associations with LM (*r* = 0.371, *p* < 0.01), BPNF (*r* = −0.236, *p* < 0.01), and BPE (*r* = −0.216, *p* < 0.01) were weak. BPNS was moderately correlated with LM (*r* = 0.468, *p* < 0.01) and PECS (*r* = 0.684, *p* < 0.01), while the remaining significant correlations were weak. LM was not significantly correlated with BPE (*r* = 0.000, *p* > 0.05). Overall, the correlations were in the expected directions and provided preliminary support for the hypothesized relationships among the study variables.

**Table 3:** Descriptive statistics and bivariate correlations among the main study variables.

Variables	*M*	*SD*	1	2	3	4	5
TLB	4.43	0.52	1				
BPNS	4.00	0.70	0.637**	1			
BPNF	2.19	0.97	−0.236**	−0.224**	1		
LM	4.42	0.60	0.371**	0.468**	0.127**	1	
PECS	6.80	1.02	0.609**	0.684**	−0.358**	0.516**	1
BPE	2.22	1.28	−0.216**	−0.257**	0.377**	0.000	−0.286**

TLB = Teacher Leadership Behavior; LM = Learning Motivation; PECS = Physical Education Class Satisfaction; BPE = Boredom in Physical Education; BPNS = Basic Psychological Need Satisfaction; BPNF = Basic Psychological Need Frustration. **p < .01 (two-tailed).

### 4.3 Common method bias test

Harman’s single-factor test was used to conduct a preliminary assessment of common method bias. The results showed that 13 factors with eigenvalues greater than 1 were extracted, and the first unrotated factor accounted for 40.993% of the total variance. Because no single factor accounted for the majority of the variance, common method bias did not pose a significant threat to the validity of this study (Kock, 2020). Additionally, a descriptive comparison between teacher self-ratings and student ratings showed that the two sets of scores displayed generally similar patterns across the five dimensions of leadership behavior. Student ratings ranged from 4.31 to 4.57, whereas teacher self-ratings ranged from 4.00 to 4.33. Because the teacher sample size was small (*n* = 6), these results are reported only as supplementary descriptive information and are not interpreted inferentially.

### 4.4 Mediation model

**Figure 2:**
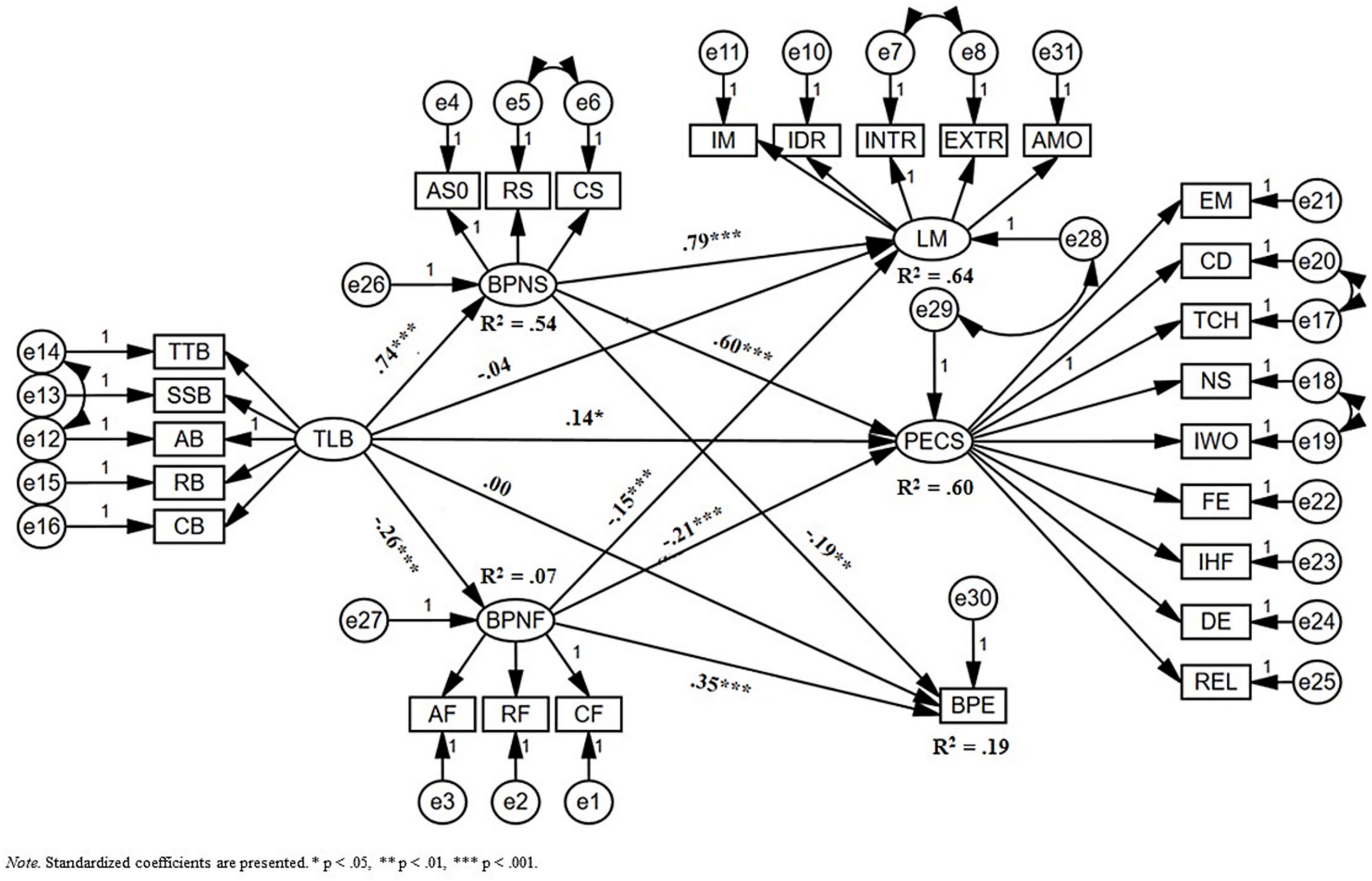
Standardized path estimates for the structural model linking PE teachers’ leadership behaviors, psychological needs, and learning experiences.

Prior to testing the mediation model, confirmatory factor analyses and higher-order factor analyses were conducted for all scales. The results indicated that the measurement structures of PE teacher leadership behavior, basic psychological need satisfaction, and basic psychological need frustration were relatively stable; therefore, these constructs were retained as latent variables in the structural model. In contrast, if learning motivation and PE class satisfaction were further specified as item-level or higher-order latent variables in the full model, the overall model complexity increased substantially and model fit deteriorated. Accordingly, the present study adopted a more parsimonious structural model in which learning motivation, PE class satisfaction, and class boredom were treated as observed variables. As illustrated in Figure 2 and Table 4, the structural model demonstrated an acceptable fit to the data (*χ^2^*/*df* = 4.087, GFI = 0.843, IFI = 0.948, CFI = 0.948, TLI = 0.940, RMSEA = 0.078). Therefore, the model was considered appropriate for subsequent mediation analyses (Kline, 2023).

**Table 4:** Direct, indirect, and total effects within the structural equation model.

Effect type	Pathway	Effect size	Boot SE	Lower limit	Upper limit	*p*
Total effect	TLB → LM	17.848	2.697	13.204	24.036	<0.001
	TLB → PECS	81.525	11.666	61.635	107.878	<0.001
	TLB → BPE	−1.052	0.262	−1.593	−0.562	0.001
Direct effect	TLB → LM	2.734	2.699	−2.330	8.461	0.275
	TLB → PECS	27.093	11.174	6.742	50.510	0.010
	TLB → BPE	−0.064	0.298	−0.652	0.511	0.832
Total indirect	TLB → LM	20.581	2.606	16.206	26.520	<0.001
	TLB → PECS	108.618	12.412	87.408	135.307	<0.001
	TLB → BPE	−1.117	0.243	−1.612	−0.669	<0.001
Via BPNS	TLB → BPNS → LM	17.501	2.678	12.822	23.505	<0.001
	TLB → BPNS → PECS	73.171	11.206	53.906	98.383	<0.001
	TLB → BPNS → BPE	−0.616	0.251	−1.143	−0.146	0.012
Via BPNF	TLB → BPNF → LM	0.347	0.376	−0.335	1.177	0.279
	TLB → BPNF → PECS	8.354	2.460	4.591	14.501	<0.001
	TLB → BPNF → BPE	−0.437	0.109	−0.696	−0.265	<0.001

TLB = teacher leadership behavior; LM = learning motivation; PECS = physical education class satisfaction; BPE = boredom in physical education; BPNS = basic psychological need satisfaction; BPNF = basic psychological need frustration.

The results showed that the overall effects of PE teacher leadership behaviors had significant and strong to very strong positive impacts on students’ learning motivation (ES = 17.8) and class satisfaction (ES = 81.5) respectively. In contrast, PE teacher leadership behaviors had a small but significant negative effect on students’ boredom. After controlling for the mediators, the direct effects of PE teacher leadership behaviors on learning motivation and boredom were no longer significant, however the effect on class satisfaction remained significant. Lastly, the total indirect effects of PE teacher leadership on all outcome variables were significant suggesting positive (learning motivation and class satisfaction) and negative mediation (boredom).

For the mediation path via BPNS, results showed significant and strong positive effects on learning motivation and class satisfaction and significant but negative effect on boredom. The results for the mediation path via BPNF showed significant positive and negative effects on class satisfaction and boredom, respectively. However, the effect of BPNF on learning motivation was not significant.

## 5 Discussion

This study examined how physical education (PE) teacher leadership behaviors are associated with students’ class-related experiences (learning motivation, class satisfaction, and class boredom), with a particular focus on the mediating mechanisms of basic psychological need satisfaction and need frustration.

The present study demonstrated that, within university compulsory PE classes, PE teacher leadership behavior did not directly predict students’ learning motivation, class satisfaction, or boredom. Instead, its effects operated primarily through two indirect pathways which are the basic psychological need satisfaction and the basic psychological need frustration. Moreover, the basic psychological need satisfaction was found to exhibit the strongest effect. That is, basic psychological need satisfaction is the main mediator explaining the relationships between teacher leadership behaviors and student learning outcomes. This finding indicates that students’ learning experiences depend less on teacher behavior per se and more on how students interpret and experience the classroom environment through their needs for autonomy, competence, and relatedness. This result is consistent with the central tenet of SDT that social environments influence individual functioning through psychological need-based mechanisms (Ryan and Deci, 2020). In other words, the influence of PE teacher leadership behavior on student outcomes appears to occur primarily through the psychological meaning it confers upon the classroom environment rather than through direct behavioral effects alone. This interpretation is also broadly consistent with Vansteenkiste et al.’s (2020) account of need-supportive processes, as well as with findings reported in educational and PE contexts by Reeve and Cheon (2021), White et al. (2021), and Tian and Shen (2023).

These findings extend prior SDT-based research by demonstrating that, within the context of Chinese university compulsory PE classes, PE teacher leadership behavior influences students’ learning experiences through the dual pathways of “need satisfaction–positive outcomes” and “need frustration–negative outcomes,” with the former playing a more prominent role. By examining these relationships in Chinese university PE classes and simultaneously incorporating both positive and negative learning outcomes, the present study extends the existing literature on PE teacher leadership and student learning experiences. This pattern aligns with the findings of Koka et al. (2021), Moreno-Murcia et al. (2018), and White et al. (2021). One plausible explanation is that the participants were primarily non-PE majors enrolled in compulsory PE classes. In this type of course, teachers’ supportive instruction, effective communication, and positive recognition may be more conducive to fostering positive classroom experiences (Tian and Shen, 2023), but may not be sufficient to elicit pronounced frustration responses.

Another noteworthy finding was that when basic psychological need satisfaction and need frustration were included in the model, the direct effects of PE teacher leadership behavior on learning motivation and boredom became non-significant but not class satisfaction, whereas the indirect effects of PE teacher leadership on all outcome variables remained significant. This finding highlights the role of BPNS and BPNF as main mediators between teacher leadership behaviors and student outcomes and further underscores the centrality of students’ psychological need states in explaining how teacher behaviors influence learning outcomes. This is highly consistent with the mediating role of need-based processes emphasized by Ryan and Deci (2020) and Vansteenkiste et al. (2020). It also highlights the value of integrating the MML with SDT. The former elucidates what teachers do in the classroom, whereas the latter clarifies how and why these behaviors affect student outcomes. Therefore, the present study contributes to the literature by integrating leadership and motivational perspectives into a unified framework for understanding students’ learning experiences in PE settings. This integrative perspective also aligns with the research orientations of Reeve and Cheon (2021) and Tian and Shen (2023). Meanwhile, class satisfaction only showed partial mediation suggesting that students’ satisfaction in PE is influenced by both their psychological need status and the teaching behaviors of their PE teacher.

Interestingly, PE motivation did not significantly correlate with boredom, implying that academic boredom in this context is not merely the absence of motivation but rather, actively shaped by contextual nuances such as task design, class pacing, and the relational instructional atmosphere. This finding corroborates other evidence that targeted leadership strategies are essential for counteracting classroom boredom and burnout through the active cultivation of a prosocial and supportive relational climate (Fang, 2025). This result also supports the perspectives of Pekrun and Goetz (2024) and Santana-Monagas and Núñez (2024) regarding academic boredom and classroom emotional experiences.

From a practical standpoint, these findings underscore that optimizing students’ learning experiences in higher education PE requires going beyond traditional instructional delivery and basic classroom management. Teachers must also enhance students’ motivation and satisfaction while reducing boredom by adopting leadership behaviors that support autonomy, strengthen competence, and foster relatedness, consistent with Reeve and Cheon’s (2021) recommendations on supportive teaching behaviors. More specifically, PE teachers should emphasize clear instruction, constructive feedback, respectful communication, and timely recognition of students’ effort and progress, as these behaviors represent important conditions for promoting need satisfaction and positive classroom experiences (Kim and Cruz, 2024; White et al., 2021). Given the activity-based, interactive, and safety-sensitive nature of PE classes, these leadership behaviors may be particularly important in physical education compared with many traditional academic subjects. For non-PE majors in particular, the interpersonal climate of the classroom and the quality of motivational support may be critical conditions for transforming passive participation into active and sustainable engagement in physical activity, a point also supported by the findings of Tian and Shen (2023), Milton et al. (2025), and Huang and Jeong (2025).

## 6 Theoretical contributions

First, this study integrates the MML and SDT, demonstrating that the relationship between PE teacher leadership behavior and students’ learning experiences is not merely a simple direct association but is better understood through the psychological mechanism of basic psychological needs. Second, it distinguishes between the two pathways of need satisfaction and need frustration, thereby providing a more nuanced explanation of the different processes through which teacher leadership behavior influences students’ positive and negative classroom experiences. Finally, this integrated framework was tested in the context of university compulsory PE classes in China, extending the applicability and explanatory power of these theories in non-specialized and institutionally structured PE courses.

## 7 Conclusion

The present study demonstrated that PE teacher leadership behavior influences university students’ learning experiences through the dual pathways of basic psychological need satisfaction and need frustration, with the need satisfaction pathway exerting a stronger effect. Learning motivation and class boredom were fully mediated, whereas class satisfaction was partially mediated. These findings support the integrated framework combining the MML and SDT, and suggest that supportive teacher leadership plays a critical role in enhancing students’ experiences in university compulsory PE classes (Ryan and Deci, 2020; Reeve and Cheon, 2021; Vasconcellos et al., 2020).

## 8 Implications

In the context of university compulsory PE classes for non-PE majors, physical education should focus not only on skill instruction but also on supporting students’ basic psychological needs. Teachers should foster students’ needs for autonomy, competence, and relatedness through clear instructional goals, appropriate task design, constructive feedback, and respectful communication. This need-supportive approach may be especially important for non-PE majors, whose prior sport experience and learning motivation often vary considerably (Reeve and Cheon, 2021). At the same time, teachers should seek to minimize controlling language, negative evaluation, and indifferent interactions to reduce students’ need frustration and classroom boredom (Curran and Standage, 2017). Furthermore, teachers may adopt more flexible instructional organization and differentiated feedback strategies based on students’ prior learning backgrounds and classroom responses, thereby strengthening their sense of participation and belonging.

## 9 Limitations and future directions

This present study is not without limitations. First, this study employed a cross-sectional self-report questionnaire design. Hence, the findings primarily reflect associations among variables and do not permit strict causal inference (Kline, 2023). Although Harman’s single-factor test was used as a preliminary assessment of common method bias, the data were collected from the same source at a single time point, and thus the potential influence of common method bias cannot be completely excluded (Jordan and Troth, 2020). Second, the sample was drawn primarily from non-PE majors enrolled in university compulsory PE classes in China. Consequently, the findings are most applicable to this specific instructional context, and caution is warranted when generalizing them to students taking PE courses in other countries, PE majors or specialized sport training settings.

Future research may adopt longitudinal designs to further examine the dynamic relationships among PE teacher leadership behavior, basic psychological needs, and learning experiences. It would also be valuable to expand the sample to include both PE majors and non-PE majors, and to further test the stability of the model across different student groups and classroom contexts.

## Data Availability

The raw data supporting the conclusions of this article will be made available by the authors, without undue reservation.
